# Retrospective study on effectiveness of Activ L total disc replacement

**DOI:** 10.1186/s13018-020-02116-4

**Published:** 2021-01-04

**Authors:** Wenzhi Sun, Peng Wang, Hailiang Hu, Chao Kong, Yong Hai, Shibao Lu

**Affiliations:** 1 Department of Orthopedics, Xuanwu Hospital of Capital Medical University, 45 Changchun Street, Xicheng, Beijing, 100053 People’s Republic of China; 2 Department of Orthopedics, Beijing Chaoyang Hospital, Capital Medical University, 8 GongTiNanLu, Chaoyang, Beijing, 100020 People’s Republic of China

**Keywords:** Total disc replacement, Sagittal alignment, Activ L

## Abstract

**Background:**

The effectiveness of lumbar total disc replacement (TDR) with different prostheses for sagittal alignment has been reported previously. However, there are only few reports on Activ L TDR and no specific evidence regarding whether sagittal alignment affects the clinical outcomes.

**Methods:**

Eighty-seven patients who underwent mono- or bi-segmental lumbar TDR with Activ L were studied. The films of the upright anteroposterior and lateral spine in neutral, flexion, and extension positions were obtained before surgery and at 1 month and 1 and 3 years after surgery. The radiographic parameters such as lumbar lordosis (LL), index level lordosis (IL), pelvic incidence (PI), pelvic tilt (PT), segmental lordosis (SL), and sacral slope (SS) were measured based on the lateral upright radiographs. Clinical outcomes were evaluated using the Oswestry Disability Index (ODI) and visual analog scale (VAS) pre- and post-operatively.

**Results:**

Eighty-seven patients with complete radiographic data were available for a 3-year follow-up period. Of these, 66 received a single-level TDR, and 21 received a 2-level TDR. At 1 month, the mean LL was similar to the pre-operative data and then was significantly increased to 45.1° at 3 years. On average, the IL tended to significantly increase, while the mean SL at L–5 was increased from 16.5° pre-operatively to 21.0° at 3 years. The mean SL at L1–2, L2–3, L3–4, and L5–S1; PI; PT; and SS showed no obvious difference after 3 years. In contrast, VAS and ODI scores showed significant improvement after surgery.

**Conclusions:**

Activ L TDR showed a favorable effect on sagittal alignment, enhancing the IL while preserving the LL and SS. However, satisfactory clinical results for over a 3-year follow-up were not affected by sagittal alignment.

## Background

For over the past few years, spinal fusion has been regarded as the standard surgical treatment for painful lumbar degenerative disc disease (DDD) due to failed conservative treatments. However, this approach eliminates the motion of the index segment and creates abnormal biomechanical conditions that might lead to the degeneration of adjacent segments, and thus requiring subsequent reoperation [1, 2]. To overcome this problem, non-fusion technologies have been developed, such as total disc replacement (TDR), and this is one of the alternate methods that can restore and maintain normal segmental motion without affecting any adjacent segments. Compared with spinal fusion, the other fundamental theoretical advantage of TDR is that it assists in restoring or maintaining the sagittal balance of the spine [3, 4].

These advantages of TDR might be the main reasons for the achievement of good clinical outcomes. However, few studies have reported conflicting results regarding the clinical outcomes. Siepe et al. [5] have prospectively analyzed the clinical efficacy of TDR with ProDisc II (DePuySynthes Inc., West Chester, PA, USA), and the results revealed satisfactory and maintained mid- to long-term clinical outcomes after a mean follow-up of 7.4 years. The midterm results of TDR with SB Charité III (DePuySynthes Inc.) prosthesis [6] and clinical results of the 1- to 3-year follow-up with Activ L artificial disc (Aesculap AG & Co. KG, Tuttlingen, Germany) [7] have been reported previously and revealed satisfactory clinical outcomes in both patient cohorts. However, a 5-year, prospective, randomized multicenter study showed no significant differences in the clinical outcomes between TDR and fusion [8]. In addition, Putzier et al. [9] have reported that long-term results of TDR implantation in DDD are at least as good as fusion; results are still lacking.

Though the relationship between the range of motion and clinical outcomes has been reported in various studies [10, 11], there are very few reports that focus on the correlation between sagittal parameters and clinical outcomes after TDR [12].

Hence, in this study, we aimed to evaluate the change of lumbar sagittal alignment over time after TDR with Activ L and determine whether sagittal alignment affects the clinical outcomes.

## Materials and methods

This is a retrospective study of 98 patients who underwent TDR using the Activ L artificial disc in our hospital from March 2009 to March 2018. Of these, 87 patients had complete radiographic data and were available for a follow-up period of 3 years.

The inclusion and exclusion criteria of patients who are eligible to receive a prosthesis in this study were carried out as described previously [7].

Indications for surgery included patients aged 25–60 years; diagnosed with DDD (low back pain of discogenic origin, with consistent symptoms of primarily back pain and, in some cases, leg pain without overt nerve root compression); absence of facet joint arthrosis confirmed by CT; with disc herniation that could be removed through anterior approach; and had unsuccessful conservative therapy of at least 6 months duration.

The exclusion criteria for surgery included patients aged > 60 years, spinal stenosis requiring decompression, spinal spondylolisthesis or instability of the spine, major spinal deformity, severe osteoporosis, compromised vertebral body, previous spinal infection or tumor, moderate or worse facet joint arthritis, and major segmental instability.

### Surgical device and technique

The Activ L total disc arthroplasty system (Aesculap AG & Co. KG, Tuttlingen, Germany) used in this study involves a modular design, offering the lowest profile of 8.5 mm. This prosthesis is a metal-on-plastic device, with three modular components: inferior and superior cobalt-chrome-molybdenum plates with either a large central keel or anterior spikes, and an ultrahigh molecular weight polyethylene (UHMWPE) inlay lying inside the inferior plate, which was similar to the Prodisc-L (DePuySynthes Inc.). This also allows a sagittal translational movement of the center of rotation (COR) from the center to the posterior end, i.e., the COR in Activ L can move from the center of up to 2 mm dorsally based on the shear forces [13, 14].

The surgical procedure was carried out as described previously [7], and the anterior retroperitoneal approach was used. Briefly, the patient was placed in a supine position on a folding operation table, and a complete discectomy through an anterior approach was performed using lumbar surgical instruments. For patients with a herniated disc, complete decompression was performed for relieving the symptoms. Care was taken to preserve the peripheral annulus fibrosis when performing a discectomy, providing ligamentotaxis. Before fitting the prosthesis, the disc space was prepared in an identical manner to ensure proper positioning of the prosthesis. Next, the anterior longitudinal ligament and the anterior annulus fibrosis were resected. The posterior longitudinal ligament was stretched to facilitate the restoration of normal disc space height. The bony vertebral endplates were left intact and were shaped to be parallel. The subchondral bone on the vertebral endplates was preserved in order to provide stability and prevent the impact of implant subsidence. A flat surface was created to maximize the bone-implant contact area. The final positioning was assessed using fluoroscopy, and the anterior longitudinal ligament was not fixed to avoid heterotopic ossification.

### Radiographic and clinical evaluation

The patients were evaluated radiographically and clinically pre-operatively and at 1 and 12 months post-operatively. All the patients were then informed to provide consent for additional follow-up every 12 months for the next 2 years.

**Fig. 1:**
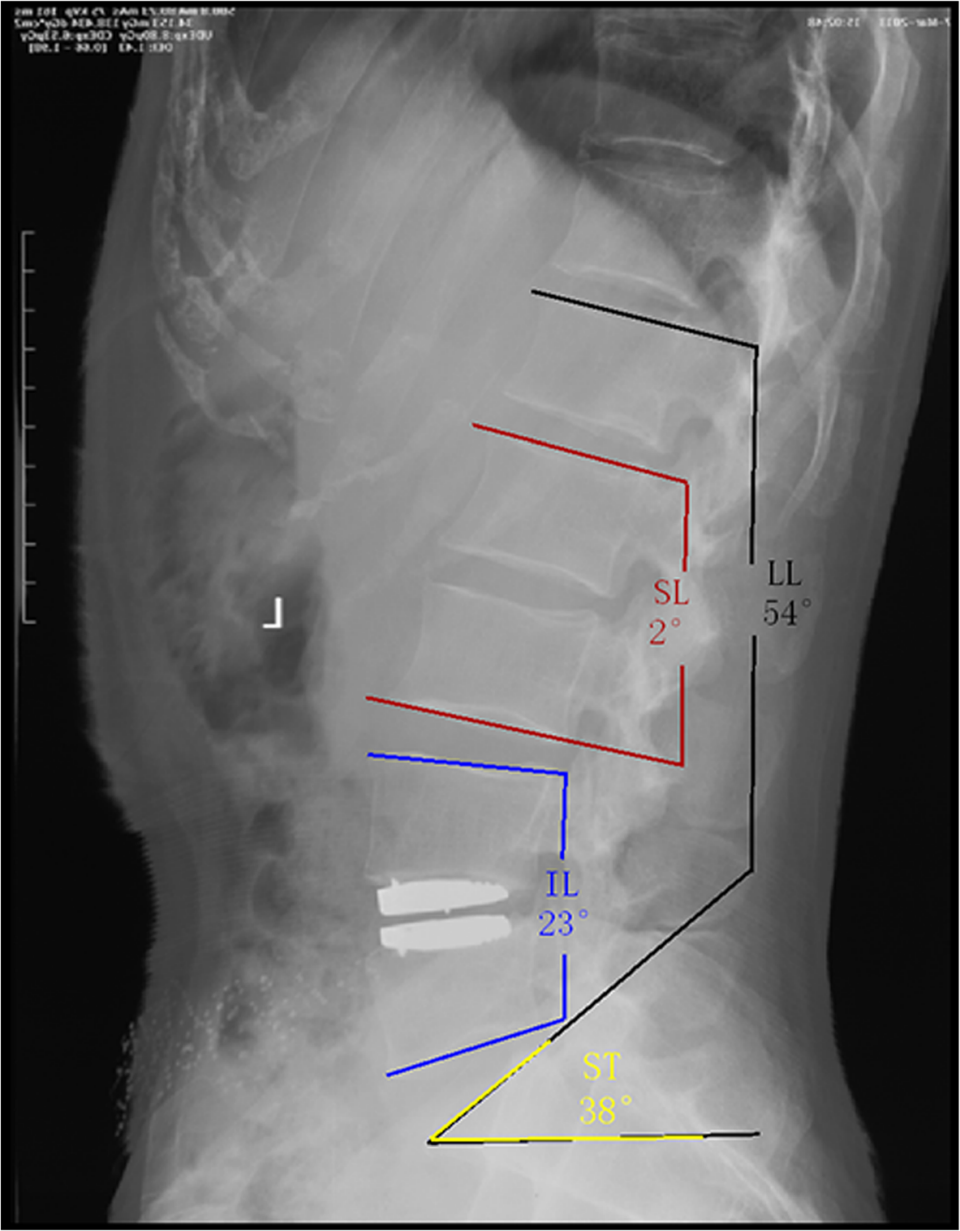
Methods for measuring the sagittal parameters

Radiographic evaluation of upright anteroposterior and lateral spine films in neutral, flexion, and extension positions before and after the operation was performed. Radiographic measurements were performed using Surgimap™ (Surgimap Spine Software, Nemaris Inc., NY, USA) [15]. The lateral upright radiographs were used to measure the sagittal parameters including lumbar lordosis (LL), index level lordosis (IL), pelvic incidence (PI), pelvic tilt (PT), segmental lordosis (SL), and sacral slope (SS). LL was calculated by the angle formed between the superior endplate of L1 and the sacral endplate. IL was calculated from the angle between the cephalad endplate of the superior vertebra and the caudal plate of the inferior vertebra at the operated level (the superior endplate of L5 and the sacral endplate were used at L5–S1). Similarly, the SL was calculated at each level from L1 to S1. The SS was calculated from the angle between the sacral endplate and the horizontal plane (Fig. 1).

The clinical outcomes were assessed by using a 10-point visual analog scale (VAS), in which the scores represent “no pain” (0 points) to “intolerable pain” (10 points), for low back and leg pain and the Chinese version of the Oswestry Disability Index (ODI) [16] for lumbar function. Post-operative controls and examinations were performed by two independent observers who are not involved in pre-operative decision-making by using the same system separately.

### Statistical analysis

Continuous data are expressed as means ± standard deviation. Repeated measures analysis of variance was used to analyze the changes in patient values. Spearman’s correlation coefficient (a multivariate logistic regression model) was used to determine the correlation between the parameters at 3-year follow-up. All statistical analyses were conducted using SPSS version 19.0 (SPSS Inc., Chicago, IL, USA), and a *P* value of less than 0.05 was considered to be statistically significant.

## Results

### Patient demographics

All the 98 patients received lumbar TDR surgery between March 2009 and March 2013. Of these, 87 (48 males and 39 females) with complete radiographic data were enrolled in this study. The mean age of the patients was 44.2 years (range, 32–54), and 66 patients received a single-level TDR and 21 received a 2-level TDR: 3 cases at L3/4, 48 cases at L4/5, 15 cases at L5/S1, 3 cases at L3/4 + L4/5, 18 cases at L4/5 + L5/S1. The details of patients’ demographics were illustrated in Table 1.

**Table 1:** Patient demographics

Patient demographics	
Sample size	87
Age (years)	44.20 ± 10.14
Male/female	48/39
Body mass index	24.34 ± 4.12
Pre-operative ODI, %	40.79 ± 1.80
Pre-operative VAS (back)	7.21 ± 0.69
Pre-operative VAS (leg)	7.15 ± 0.83
**Affected segment**	
L3–4	3
L4–5	48
L5–S1	15
L3–L5	3
L4–S1	18

### Radiographic parameters

The values of LL, PI, PT, IL, SS, and SL at each level at different time points are shown in Table 2.

**Table 2:** Radiographic parameters at different time points

	Pre-op	1 month Post-op	1 year Post-op	3 years Post-op	*F*	*P*
LL	40.21 ± 2.60	37.07 ± 1.82	43.31 ± 2.10	44.41 ± 2.10	5.258	0.003
PI	49.79 ± 7.20	49.59 ± 7.14	50.34 ± 7.67	50.24 ± 7.48	2.161	0.107
PT	16.00 ± 8.30	15.24 ± 7.26	13.86 ± 7.03	14.86 ± 7.91	0.657	0.558
IL	18.66 ± 1.86	23.07 ± 1.07	25.41 ± 1.71	25.93 ± 1.69	19.728	< 0.001
SS	33.79 ± 1.92	34.35 ± 1.78	36.48 ± 1.66	35.38 ± 1.55	1.182	0.320
SL (L2–3)	0.41 ± 0.71	− 0.41 ± 0.66	− 0.21 ± 0.70	0.48 ± 0.67	2.105	0.116
SL (L3–4)	6.48 ± 1.03	5.62 ± 0.97	7.00 ± 1.09	7.41 ± 0.99	2.438	0.083
SL (L4–5)	16.52 ± 1.53	17.86 ± 1.40	20.35 ± 1.32	21.03 ± 1.33	7.768	< 0.001
SL (L5-S1)	19.10 ± 1.49	19.69 ± 1.07	20.79 ± 1.28	21.28 ± 1.23	1.421	0.250

*Pre-op* pre-operative, *Post-op* post-operative, *LL* lumbar lordosis, *PI* pelvic incidence, *PT* pelvic tilt, *IL* index level lordosis, *SS* sacral slope, *SL* segmental lordosis

### Lumbar lordosis

The values of LL after surgery were significantly increased (*P* = 0.003). Although no obvious differences at 1-month follow-up were observed when compared with the pre-operative values (*P* = 1.000), the values at 1-month follow-up were increased from 37.07 ± 1.82 to 43.31 ± 2.10° at 1-year follow-up (*P* = 0.002). Also, the values showed a significant increase at 3-year follow-up when compared with 1-month follow-up (*P* = 0.017). However, at 1-year and 3-year follow-up evaluations, the LL values showed no significant differences when compared with the pre-operative data (*P* = 0.996, *P* = 0.397).

### Index level lordosis

The average values of IL showed an increased tendency (*P* < 0.001). The pre-operative values and those values at 1-month, 1-year, and 3-year follow-up showed significant differences (*P* = 0.007, *P* < 0.001, *P* < 0.001, respectively). The values were increased from 23.07 ± 1.07° at 1-month follow-up to 25.41 ± 1.71° at 1 year post-operatively (*P* = 0.005). However, the values of IL showed no significant differences over the follow-up period between 1 month and 3 years (*P* = 0.053), and the values between 1-year and 3-year follow-up also showed no significant differences (*P* = 1.000).

### Sacral slope

The SS was not significantly affected by the operation. The mean value of SS showed no significant difference at any time point evaluated (*P* = 0.320).

### Segment lordosis

In general, the SL at the L4–5 level was increased after surgery (*P* < 0.001), but the differences between pre-operative SL values at the L4–5 level and the values at 1 month after surgery showed no significant differences (*P* = 1.000). At 1 year and 3 years after surgery, the values were increased significantly when compared with pre-operative values (*P* = 0.008, *P* = 0.016) and 1 month values (*P* = 0.018, *P* = 0.029). However, the values at the L4–5 level at 3-year follow-up showed no significant differences when compared with those at 1 year (*P* = 1.000). Analysis of the changes at L1–2, L2–3, L3–4, and L5–S1 levels showed no significant differences (*P* = 0.463, *P* = 0.116, *P* = 0.083, *P* = 0.250, respectively).

### Clinical results

**Fig. 2:**
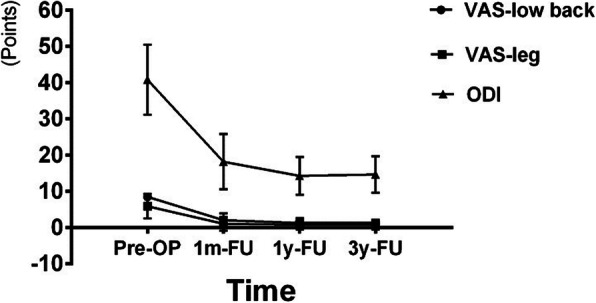
Clinical outcomes. Measured by VAS for low back and leg pain and ODI pre-operation and at follow-up visit (i.e., at 1 month, 1 year, and 3 years)

The mean back pain of the patients was decreased significantly after surgery, and the situation was well maintained during the follow-up time. Compared with the pre-operative values, the mean VAS scores for low back were significantly decreased at 3-year follow-up (*P* < 0.001, Fig. 2). The changes in the mean leg pain showed a similar trend (Fig. 2). The pre-operative ODI was 40.79 ± 1.80 and was 14.62 ± 0.93 at 3-year follow-up (*P* < 0.001, Fig. 2).

A total of 87 patients achieved satisfactory clinical results, and 60 patients went back to their original work, and 27 patients have changed jobs. When enquired if they would choose the same TDR surgical treatment again, 75 patients declared a positive intention (86.2%), 12 answered “not sure” (13.8%), and no one declined.

### Correlation of sagittal parameters and clinical outcomes

The correlation between sagittal parameters and clinical outcomes was measured by Spearman’s correlation coefficient. At 3-year follow-up, the results showed that neither of the sagittal parameters studied demonstrated any correlation with VAS for low back and leg pain nor ODI score (Table 3).

**Table 3:** Significant values for sagittal parameters and clinical outcome

	VAS (back pain)	VAS (leg pain)	ODI
LL	0.194	0.182	0.729
PI	0.079	0.435	0.751
PT	0.237	0.603	0.622
IL	0.403	0.683	0.468
SS	0.593	0.593	0.855
SL (L1–2)	0.614	0.842	0.146
SL (L2–3)	0.222	0.856	0.318
SL (L3–4)	0.252	0.356	0.753
SL (L4–5)	0.445	0.656	0.956
SL (L5–S1)	0.275	0.710	0.721

*LL* lumbar lordosis, *PI* pelvic incidence, *PT* pelvic tilt, *IL* index level lordosis, *SS* sacral slope, *SL* segmental lordosis, *VAS* visual analog scale, *ODI* Oswestry Disability Index

### Complications

There was no device failure or the occurrence of any major complications, and there was any evidence of neurological deterioration or requirement for revision surgery during the follow-up period. Adjacent segment disease (ASDis) was found in one patient who complained of residual low back pain (VAS 5) and leg pain (VAS 4) at 3 years after surgery, and the ODI score was 20 points. The patient refused an MRI or CT scan or reoperation due to pain was relieved by non-steroidal anti-inflammatory drugs. Prosthesis subsidence was observed in 3 patients (with measurement of 3.2 mm, 4.1 mm, and 3.6 mm), but no symptoms were seen. Heterotopic ossification was observed in one patient at 36 months post-operatively, but no symptoms were reported. Among all the male patients, no retrograde ejaculation was observed.

## Discussion

Although spinal fusion is still regarded as the most common treatment for symptomatic DDD, it is unresponsive to conservative treatment modalities, as persistent post-fusion pain is a common and refractory problem. Postfusion pain is independent of factors such as poor posture, paraspinal muscle pain, or pseudoarthrosis and showed a significant correlation with sagittal imbalance such as decreased SS, increased pelvic tilt, and decreased LL [17].

Our previous study on better treatment options in patients with DDD has led us to the discovery of a more preferable option of motion-preserving total disc arthroplasty [6, 7], avoiding fusion. But to our knowledge, the effect of TDR with Activ L on sagittal alignment has not been studied to date. A study conducted by Kasliwal and Deutsch [18] reported a significant and near-significant increase in the trend of IL and LL, respectively, after TDR using Charité and Activ L artificial discs with a follow-up time ranging from 12 to 24 months. Another study conducted by Chung et al. [19] with a mean follow-up period of 30 months showed a significant increase in LL in all patients who underwent TDR using ProDisc without any significant changes in SS or pelvic tilt during the latest follow-up visit. In patients with single-level TDR, the mean SL at L5–S1 and L4–5 operative levels showed a significant increase. But Cakir et al. [12] have reported that TDR with ProDisc showed a significant increase in IL, while LL is unchanged during a mean follow-up period of 15.3 months (range, 12–35). In addition, Le Huec et al. [4] have reported that patients were able to maintain their pre-operative sagittal balance after TDR, and these changes of sagittal parameters showed no significant differences between the pre-operative values and those at the last post-operative follow-up visit. According to these studies, the prosthesis led to enough freedom of motion, which allows the patient to maintain a natural sagittal and spinopelvic balance to prevent the potential undue stress on the muscles as well as on the sacroiliac joint. In our study, LL and SL at the L4–5 level showed no obvious difference at 1 month when compared to those pre-operatively. However, LL showed no obvious difference between the values at 1 and 3 years after surgery when compared to those of the pre-operative data, while SL at the L4–5 level showed an increasing tendency during the follow-up period. The average values of IL showed a similar increasing tendency in our study. The other sagittal parameters showed no significant changes.

There are many reasons for the differences in the results after TDR. The design of the prosthesis might play an important role in the sagittal alignment of the lumbar spine. An in vitro study conducted by Wilke et al. [3] on TDR using unconstrained Charité, semi-constrained Prodisc, and semi-constrained Prototype with a more posterior center of rotation than the Prodisc showed that all the prostheses resulted in a significant increase in IL when compared with intact status. The IL after prototype implantation was significantly lowered when compared with Charité (*P* = 0.024) or Prodisc (*P* = 0.044), while no significant difference in IL was observed between Charité and Prodisc. Furthermore, according to few study reports, there was no correlation between the angle of implant chosen and the post-operative angle of lordosis at the operated level [18], thus showing no association of LL either with the sagittal prosthesis displacement or with the prosthesis size [20]. Besides, the surgical levels might also influence the sagittal alignment. Tournier et al. [20] have found that modification of LL is linked to the operative levels, and this is because the segmental contribution of the lordotic curvature has been increased distally. This represents 93% of the L4–S1 curvature of LL after implantation of L3–4 prosthesis but was 73% of LL after implantation of L4–L5 and L5–S1 prostheses. The implantation technique chosen is also regarded as an important factor. In a study that compared anterior implantation with oblique implantation, Schmidt et al. [21] revealed that both anterior and oblique-implanted TDR showed a significant increase in IL while retaining LL, and the increase of IL was lowered in the oblique-implanted group, which was probably due to the retention of the anterior longitudinal ligament. A finite element study conducted by Dooris et al. [22] showed that the anterior longitudinal ligament assists in restoring the normal load sharing and segment stiffness in the sagittal plane. The anterior longitudinal ligament was resected during surgery, and this could be the main reason for the increased tendency of IL and SL at L4–5. Apart from all the above reasons, the follow-up period might also influence the results. As observed in this study, some parameters have been changed over time, and this is because of the relatively small sample size, and the data with regard to subsidence or heterotopic ossification were not analyzed, affecting the results of our study. To access better information on potentially influential factors, advanced examinations such as MRI and computed tomography scans should be included during the follow-up period. Besides, the femoral head is not in the field of vision of lateral spine films before surgery. Therefore, we could not measure the sagittal alignment parameters such as pelvic incidence and pelvic tilt. And no full-length radiographs of the spine were taken before surgery, then the sagittal vertebral axis could not be measured.

Finally, the purpose of all interventions involves the achievement of a good clinical outcome. Clinical results of this research are similar to those of our previous study on the outcomes of TDR with Activ L [7]. The correlation between sagittal alignment and clinical outcomes after fusion has been studied in previous studies [17, 23]. To our knowledge, only Cakir et al. [12] have reported the relationships between the two after TDR. The study also revealed that patients with physiological SL vs. unphysiological SL showed no differences in the pre- and post-operative outcome measures. In our study, with the use of an Activ L TDR, the patient is able to maintain the pre-operative sagittal balance with no significant change in any of the variables studied and most of the patients showed significant improvement of clinical symptoms. This improvement might not only be related to the replacement of degenerative painful disc and the unloading of facet joints after the restoration of disc height, but also to the restoration of favorable sagittal alignment. Allow the patient to maintain the natural sagittal needed to prevent undue stress on the muscles and the sacroiliac joint.

## Conclusions

In conclusion, the effect of Activ L TDR on sagittal alignment showed favorable outcomes, enhancing IL while preserving LL and SS. The 3-year follow-up findings showed satisfactory clinical results, and are unaffected by sagittal alignment. The effectiveness of the Activ L TDR requires further evaluation of long-term results.

## Data Availability

All data generated or analyzed during this study are included in this published article.

## Abbreviations

TDR: Total disc replacement
LL: Lumbar lordosis
IL: Index level lordosis
PI: Pelvic incidence
PT: Pelvic tilt
SL: Segmental lordosis
SS: Sacral slope
ODI: Oswestry Disability Index
VAS: Visual analog score
DDD: Degenerative disc disease
COR: Center of rotation
ASD: Adjacent segment disease
